# Role of the Coreactant on the Dual-Source Behavior of Lithium Hexamethyldisilazide for ALD Li-Containing Films

**DOI:** 10.1021/acs.jpcc.4c05987

**Published:** 2024-11-07

**Authors:** M. J. Pieters, L. Bartel, C. van Helvoirt, M. Creatore

**Affiliations:** † Department of Applied Physics and Science Education, Eindhoven University of Technology, Eindhoven 5600 MB, The Netherlands; ‡ Eindhoven Institute of Renewable Energy Systems (EIRES), PO Box 513, Eindhoven, 5600 MB, The Netherlands

## Abstract

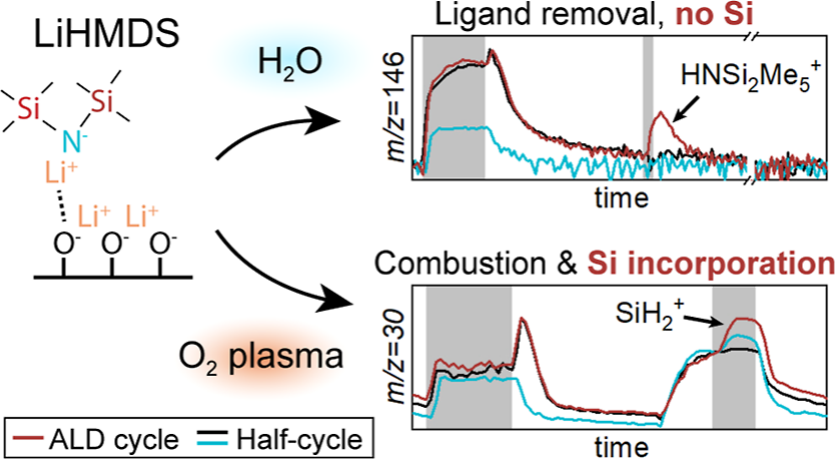

Atomic layer deposition (ALD) of Li-containing thin films is deemed as highly relevant for the development of next-generation Li-ion batteries. Lithium hexamethyldisilazide (LiHMDS), as Li-containing precursor, is preferred over the widely used lithium *tert*-butoxide because of its lower melting point of 70 °C. However, the presence of silyl groups in the LiHMDS chemical structure can result in the undesired incorporation of Si in the film. Therefore, understanding the reaction mechanism of LiHMDS is required to control its dual-source behavior and grow Si-free Li-containing thin films. For this purpose LiHMDS was combined with O_2_ plasma or water as coreactant. *In situ* spectroscopic ellipsometry (SE) and X-ray photoelectron spectroscopy (XPS) revealed that using O_2_ plasma as coreactant results in linear growth and Si-containing films, whereas using H_2_O as coreactant leads to fast, nonsurface-reaction-limited growth and Si-free films. To shed light on the role of the coreactant on the reaction mechanism of LiHMDS, *in situ* studies by time-resolved quadrupole mass spectrometry (QMS) were performed on the O_2_ plasma and H_2_O-based ALD processes. Measurements taken during full ALD cycles and half-cycles were carefully compared to identify which half-cycle surface reaction products lead to silicon incorporation in the film. The QMS results of the LiHMDS + H_2_O process showed that LiHMDS both chemisorbs and physisorbs. Furthermore, it is concluded that Si incorporation occurs during the O_2_ plasma step, when the physisorbed ligands are combusted and Si-containing products are redeposited. This work also demonstrates that the incorporated Si can be abstracted from the film by means of a H_2_ plasma step following the O_2_ plasma step. These insights on the role of the coreactant in the synthesis of Li-containing films contribute to the development of LiHMDS-based ALD processes for Li-ion battery applications.

## Introduction

The development of next-generation Li-ion batteries requires understanding of and control over interface reactions that drive both the operation and degradation of the battery. Atomic layer deposition (ALD) is a suitable tool to improve the stability of interfaces in batteries, and its use in Li-ion battery research has increased substantially over the past decade.^1^ A key example of how ALD is applied in batteries is electrode–electrolyte interface engineering. ALD delivers uniform and conformal thin films with good control over thickness and composition, and therefore films can be grown on complex electrode structures to suppress parasitic reactions and improve battery performance. These films should be electrochemically stable and permeable to Li ions. One of the most popular materials to apply on electrodes is Al_2_O_3_, which has been shown to improve the cycling stability of NMC cathodes^2^ and Li metal anodes.^3^ However, Al_2_O_3_ is a poor ionic conductor. Therefore, ALD of Li-containing films, that generally have higher ionic conductivities, is becoming more relevant. Examples of Li-based ALD materials are LiF,^4−6^ Li_2_CO_3_,^7^ Li_3_PO_4_,^8^^,^^9^ and ternary oxides such as LiAl_*x*_O_*y*_,^10,11^ LiNb_*x*_O_*y*_,^12,13^ and LiTi_*x*_O_*y*_.^14^

Additionally, ALD can be used to deposit thin film solid-state electrolytes, such as N-doped Li_3_PO_4_ (LiPON). It has been shown that ALD provides control over the N incorporation,^15−17^ and films with ionic conductivities up to 1.7 × 10^–6^ S/cm have been recently achieved.^17^ When implemented in a thin film solid state battery, it was shown that conformal and pinhole-free ultrathin (<100 nm) LiPON films can be fabricated.^15,18^

Furthermore, cathode materials can be synthesized by ALD, either in view of engineering microbatteries, or to prepare simplified cathode model systems with respect to 3D cathodes consisting of cathode particles embedded in a binder. Then, the model systems serve to gain fundamental understanding of cathode-electrolyte interface processes. ALD of ternary cathode materials, such as LiCoO_2_^19,20^ and LiMn_*x*_O_*y*_^21,22^ has been reported. It was shown that the stoichiometry of these films can be controlled by varying the subcycle ratio, and the fabricated cathode films are electrochemically active and show good reversible electrochemical performance.

When looking at ALD Li precursors, lithium *tert*-butoxide (LiO^t^Bu) has been the most widely adopted so far, but it requires high temperatures (130–180 °C) to vaporize it.^23^ Lithium hexamethyldisilazide (LiHMDS) is easier to handle because of its lower melting point at 70 °C, which implies that lower temperatures of precursor pod and dosing lines are required, and line clogging is less probable. LiHMDS has been used for the ALD growth of battery materials, such as LiF,^4,24,25^ Li_3_N,^26^ Li_2_CO_3_,^26^ Li_3_PO_4_,^9,27^ LiPON.^28,29^ However, the LiHMDS molecule contains silyl groups, which can result in the incorporation of Si in the deposited films.^30^ Since the presence of Si in the film is generally not desired, understanding the ALD reaction mechanism of LiHMDS is required to control its dual-source behavior and grow Si-free films.

Previous studies on the reaction mechanism of LiHMDS concluded that Si incorporation occurs during the LiHMDS dose step, due to competition between LiHMDS and hexamethyldisilazane (HMDS) for chemisorption on OH-surface sites.^31,32^ HMDS is formed when LiHMDS chemisorbs on OH-groups and is known to chemisorb on OH-groups itself as well.^33^ Therefore, the presence of OH-surface groups is reported to be the main origin of Si incorporation in the film. Furthermore, Werbrouck et al. proposed that LiHMDS also physisorbs, and that the combustion of physisorbed LiHMDS ligands by an O_2_ plasma (O_2_*) might be a second source of Si incorporation.^32^ Whereas some publications that combine LiHMDS with O_2_* or O_3_ report Si content values up to 24 at. %,^30,32^ others report negligible levels of Si.^34^ Interestingly, the Si levels are consistently low when the LiHMDS dose is directly followed by a H_2_O dose^12,26^ or another precursor dose, such as TMA,^27^ TMP^27,32^ or TDMAT.^35^ This suggests that the coreactant plays a bigger role in the Si incorporation mechanism than the surface termination.

In this work we show that the single- or dual-source behavior of LiHMDS can be controlled by the choice of coreactant. The effect of the coreactant is studied by combining LiHMDS with H_2_O and O_2_*. First the film growth, stoichiometry and optical constants are discussed, focusing on differences between the two ALD processes. Subsequently, the two ALD processes are further investigated by *in situ* time-resolved quadrupole mass spectrometry (QMS) to determine the reaction products formed during film growth. We adopt a measurement procedure in which the standard ALD process is compared to the processes in which only one of the two reactants is dosed. A comparison between the full ALD cycle and the half-cycles allows to investigate the differences in reaction products for different surface terminations, and discern between the presence of reaction products and pressure-induced fluctuations in the measured QMS signal. Our results show that LiHMDS both chemisorbs and physisorbs, and that redeposition of Si-containing ligand combustion products during the plasma step is the main origin of Si incorporation in the film. Furthermore, we show that Si can be abstracted from the deposited films by an additional H_2_* step added to the O_2_*-based process.

## Experimental Details

The ALD processes presented in this work were carried out using the thermal and remote plasma ALD reactor FlexAL (Oxford Instruments). The reactor is equipped with a rotary and turbo molecular pump, such that a base pressure of <10^–6^ Torr can be reached in the reactor. The pump unit and the inductively coupled plasma (ICP) source are connected to the deposition chamber through gate valves. Lithium hexamethyldisilazide (LiHMDS) (97%, Sigma-Aldrich) was used as precursor in all ALD processes in this work, and H_2_O, O_2_*, and H_2_* were used as coreactants. The LiHMDS precursor was kept in a stainless steel container at 85 °C and the supply line was heated to 120 °C to prevent precursor condensation. The precursor was dosed into the reactor using a 100 sccm Ar flow as carrier gas. A filter with 20 μm pores was placed on the Ar inlet of the bubbler to prevent the precursor from entering the Ar line. The precursor dose step was followed by a 10 or 15 s Ar purge to remove all unreacted species and reaction products from the reactor.

In the thermal ALD process H_2_O was dosed for 50 ms, after which all valves were closed for 1 s to allow for the molecules to react. This was followed by a 60 s Ar purge. In the plasma-assisted ALD processes a 3 s O_2_* (50 sccm O_2_, 300 W) was used as coreactant, either by itself or in combination with a 2 s H_2_* (50 sccm H_2_, 200 W). To ensure a stable gas flow and reactor pressure, the reactor was filled with O_2_ or H_2_ gas prior to switching on the plasma source. The O_2_* and H_2_* steps were followed by 5 and 10 s Ar purges, respectively. The Ar purge times have not been optimized, but they were chosen sufficiently long to prevent CVD contributions. All depositions were done on a Si(100) wafer with native oxide (Siegert Wafer, 10–20 ohm cm). The process table was heated to 200 °C and the wall temperature was maintained at the maximum temperature of 120 °C.

The air-sensitive films were capped with ∼5 nm of Al_2_O_3_ (40 cycles DMAI + O_2_*) before they were taken out of the ALD reactor. Air exposure of all Li-containing films in this work was minimized by storing them in a N_2_ glovebox directly after the deposition, and by transferring them in an inert atmosphere using a transfer tube.

The growth-per-cycle (GPC) and optical properties were determined using *in situ* spectroscopic ellipsometry (SE) over the wavelength range 1.2–4.7 eV with a J.A. Woollam, Inc. M2000U tool. The Cauchy formula was adopted for all films. The air-sensitivity of the (uncapped) grown films was verified by comparing the *in situ* SE measurements to *ex situ* variable angle SE (VASE) measurements performed on a J.A. Woollam, Inc. M-2000D (1.2–6.0 eV) system at angles of incidence in the range 60–80° in steps of 5°. For the LiHMDS + O_2_* film the Cauchy formula was used, for the other, air-sensitive films a B-spline model was used.

X-ray photoelectron spectroscopy (XPS) was performed using a Thermo Scientific K-Alpha+ system with monochromatic Al Kα X-rays. The spot size of the beam was 400 μm and the base pressure of the system was 10^–8^ mbar. Depth profiles were obtained using an Ar ion gun of 500 eV during 15 or 20 s for each sputter step.

A Pfeiffer Vacuum mass spectrometer with a mass-to-charge (*m*/*z*) range of 200 atomic mass units (amu) was connected to the ALD reactor through a pipeline and a 150 μm diameter pinhole. The system is equipped with a channeltron detector and the electron energy in the ionizer was set to 70 eV. The pressure in the QMS was maintained below 10^–5^ mbar by differential pumping with a turbomolecular pump. During the QMS measurements the table temperature was lowered to 120 °C to match the reactor wall temperature. Prior to each measurement the reactor walls were covered with Al_2_O_3_ (60 cycles TMA + H_2_O). The measuring time was set to 200 ms per amu. For the time-resolved measurements, selected *m*/*z* values were tracked per channel using a dwell time of 50 ms. A maximum of 5 *m*/*z* values were measured simultaneously, such that the time resolution was at least 250 ms. Measurements were taken during the standard ALD cycles, and during half-cycles, in which (at least) one of the reactants has been left out. This allows to distinguish between background signals (e.g., precursor fragmentation in the QMS, pressure-related signal) and the formation of ALD reaction products. For all processes at least 10 (half-)cycles were monitored to verify that a steady-state was achieved.

## Results and Discussion

### Influence of the Coreactant on Si Incorporation in the Film

**Figure 1:**
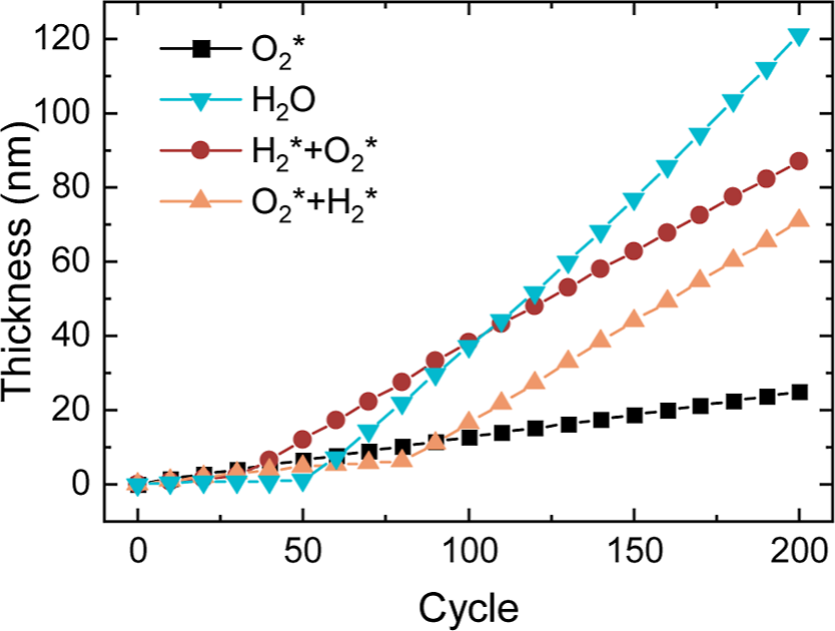
Film thickness as function of number of cycles measured by *in situ* spectroscopic ellipsometry for ALD of Li-containing films using LiHMDS combined various coreactants at a table temperature of 200 °C.

First the growth behavior and film composition of the ALD processes with LiHMDS combined with O_2_* or H_2_O as coreactants is compared. Figure 1 shows the film growth for both processes as measured by *in situ* SE at the deposition temperature of 200 °C. Additionally, the growth for processes with two subsequent plasma steps are shown, which will be discussed later on in this work. The LiHMDS + O_2_* process exhibits linear ALD-type growth. Based on the saturation curves in Figure S1 (Supporting Information) a LiHMDS dose time of 6 s and an O_2_* exposure time of 3 s were selected. The resulting growth per cycle (GPC) of 1.2 Å/cycle is comparable to the previously reported GPCs for the same process.^32,34^

Instead, the LiHMDS + H_2_O process shows two distinct growth regimes: an initial slow growth regime of 20–50 ALD cycles until about 1 nm was grown, followed by a fast growth regime with growth rates of ∼5–8 Å/cycle. Similar growth behavior has been reported for the thermal processes using LiHMDS^26^ and LiO^t^Bu,^11,36^ which is generally attributed to the hygroscopic nature of LiOH. It has been shown that LiHMDS can react with bulk OH-groups,^31^ and this nonsurface-reaction-limited growth could explain the observed high growth rates.

XPS surface scans were performed to determine the stoichiometry of the grown films. Table 1 shows that the LiHMDS + O_2_* process results in a Si content of 16.2 at. %, which is lower than the Si content of 23–24 at. % reported by Werbrouck et al. for depositions at 150 and 300 °C,^32^ but higher than the ∼1 at. % Si reported by Maximov et al. at 300 °C.^34^ These differences among various publications on the same ALD process indicate that the dual-source behavior of LiHMDS might be affected by other aspects, such as the reactor geometry or gas flows that can, for example, result in different residence times of the gas species. The Si 2p spectrum in Figure S2 shows that the incorporated Si is not fully oxidized, but may still be bonded to one or two methyl groups or hydrogen. The XPS depth profile in Figure S3 shows approximately equal Li and Si atomic percentages but no presence of N in the bulk. Therefore, the observed Si content does not originate from the incorporation of the entire HMDS ligand. 4–6 at. % F was detected on the surface of the films, but not in the bulk (see Figures S3 and S4). This surface contamination originates from the loadlock of the ALD reactor, as explained in more detail in the Supporting Information.

**Table 1:** Chemical Composition of Films Grown at 200 °C Determined From XPS Surface Measurements^a^

process	O at.% (±0.6)	Li at.% (±0.5)	Si at.% (±0.2)	C at.% (±0.2)	N at.%	Al at.% (±0.5)	F at.% (±0.2)
LiHMDS + O_2_*	48.9	25.9	16.2	4.9	<1	–	4.2
LiHMDS + H_2_O	51.7	13.2	<1	5.0	<1	24.3	5.8
LiHMDS + O_2_* + H_2_*	47.0	22.8	<1	4.1	<1	20.1	6.0
LiHMDS + H_2_* + O_2_*	47.4	23.7	<1	4.3	<1	19.6	5.1

a The films that were observed to be air-sensitive were capped with ∼5 nm Al_2_O_3_. The F is present only at the film surface (see XPS depth profiles in the Supporting Information) and originates from the scroll pump that is connected to the load lock of the ALD reactor. The errors in the atomic percentages are estimated based on variations in the range of the fitted background.

The LiHMDS + H_2_O process resulted in air-sensitive films, which is in line with previous reports on thermally grown films using LiHMDS.^26,37^ The films visibly changed upon air exposure (see Figure S5), and *ex situ* SE measurements showed that the film thickness increased significantly after air exposure (Table S1). Therefore, the air-sensitive film was capped with ∼5 nm of Al_2_O_3_ before transporting it to the XPS tool. Table 1 and the XPS depth profile in Figure S4 show that the LiHMDS + H_2_O process results in a Si-free film, which is in agreement with previous studies that showed Si-free films using a H_2_O pulse after the LiHMDS dose to grow Li_2_CO_3_^26^ and in supercycle processes of ternary oxides that contain a LiHMDS + H_2_O subcycle.^12^ Furthermore, Al is detected throughout the ∼120 nm film, which suggests that the Li-based film has a porous structure. The Al diffusion in the film shadows the exact bulk composition of the Li-based film.

The difference in Si content between the LiHMDS + O_2_* and LiHMDS + H_2_O processes strongly suggests that the dual-source behavior of LiHMDS is related to the choice of coreactant. Therefore, both ALD processes are investigated further using QMS to elucidate the reaction mechanism of LiHMDS in both processes.

**Figure 2:**
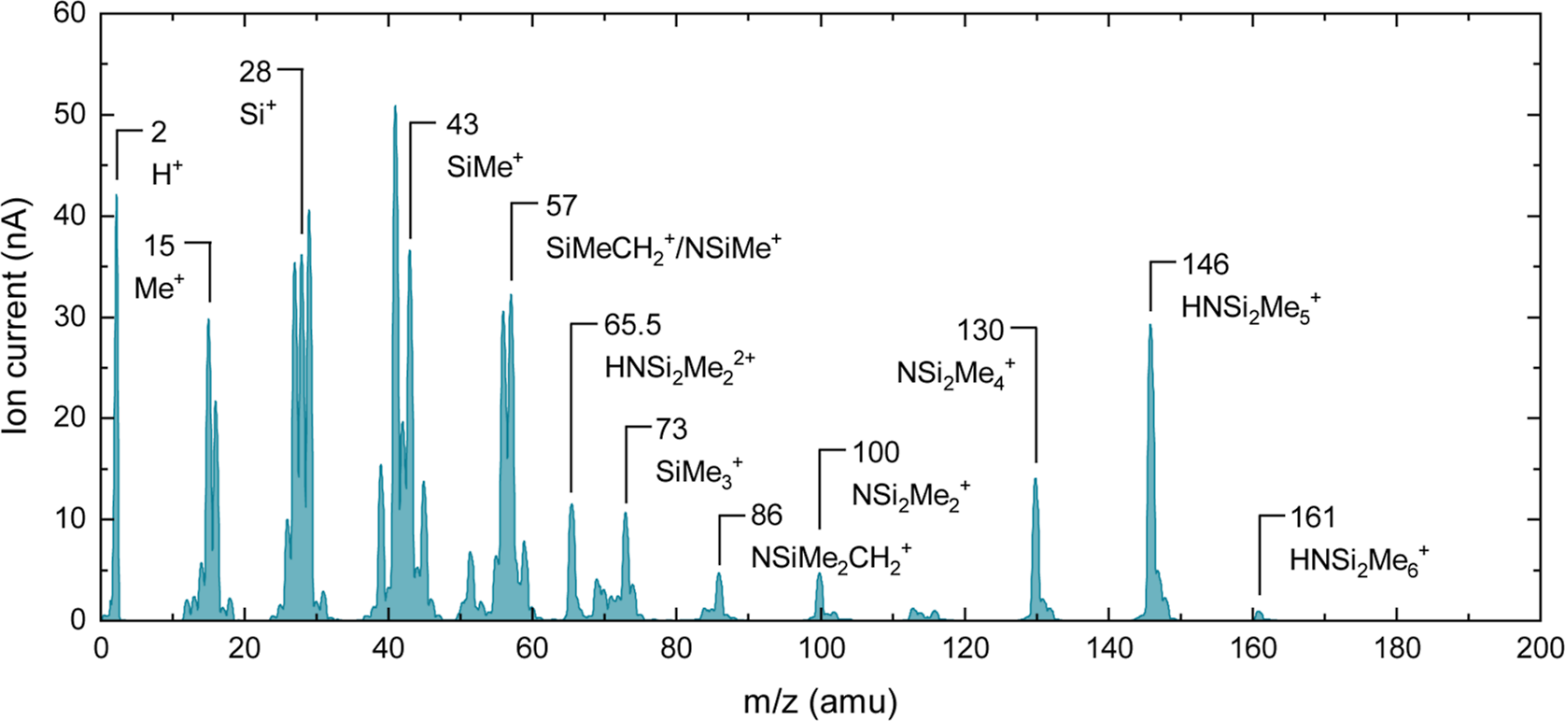
Mass spectrum of LiHMDS. LiHMDS was dosed into the reactor using the vapor-drawn method to obtain a mass spectrum free of Ar-related signals.

First, the mass spectrum of LiHMDS is measured to determine the relevant *m*/*z* ratios for the time-resolved measurements. The peaks observed in Figure 2 are in line with previously reported mass spectra of LiHMDS^4,32^ and HMDS.^38^ The peak at *m*/*z* = 161 amu is attributed to the HMDS parent ion. The LiHMDS parent ion would correspond to *m*/*z* = 167 amu, but this peak is not observed, since it is known that LiHMDS is present as a dimer in the vapor phase.^39,40^ On a logarithmic scale (Figure S6), the mass spectrum reveals a low peak at *m*/*z* = 174 amu, which has been observed before and was attributed to the Li(LiHMDS)^+^ fragment originating from the LiHMDS dimer.^41^

Several features in the mass spectrum can be attributed to the HMDS ligand with one or multiple abstracted methyl groups upon electron impact. Correspondingly, a strong peak is observed at *m*/*z* = 15 amu, attributed to CH_3_^+^. The strong peak at *m*/*z* = 146 amu is attributed to the HNSi_2_Me_5_^+^ fragment, which is formed when HMDS loses one methyl group. This is also the feature with the highest intensity in the mass spectrum of HMDS.^38^ Since HMDS is expected to be formed during the LiHMDS half-cycle, this mass is selected to track the presence of HMDS, and therefore the precursor, in the ALD reactor.

*In situ* time-resolved QMS measurements were performed to analyze the ALD reaction products. Specifically, the LiHMDS + O_2_* and LiHMDS + H_2_O processes are compared to identify the reasons for the observed difference in film growth and composition. Measurements were performed on a selection of relevant *m*/*z* values, listed in Table 2. In addition to the standard recipe (SR), QMS data are shown for half-cycles, in which either the precursor or the coreactant is absent, resulting in no film growth. In the discussion of the time-resolved QMS results, we focus on the differences between the QMS signals of the SR and the half-cycles, which provide insights into the ALD reaction products. Furthermore, the pressure in the ALD reactor was monitored to identify any pressure influence on the QMS signal. All measurements that contain a LiHMDS dose step show a spike in ion current at the onset of the precursor purge, that is also visible in the pressure curve. This spike is attributed to a pressure effect caused by the filter that is placed on the inlet of the LiHMDS bubbler. The resistance created by the filter causes a build-up of Ar in the line that is released at the start of the purge. The table temperature was lowered to 120 °C to match the wall temperature, as it is expected that most of the QMS signal will originate from the walls due to their larger surface area. It was confirmed that the GPC (1.1 Å/cycle) and film composition of the LiHMDS + O_2_* process were similar to previous depositions done at 200 °C (see Figure S3).

**Table 2:** Relevant *m*/*z* Ratios for the Studied Processes, Their Assigned Ions and Their (Main) Assigned Parent Molecules^a^

*m*/*z* (amu)	assigned ions	assigned parent species
15	CH_3_^+^	CH_4_
18	H_2_O^+^	H_2_O
30	SiH_2_^+^, NO^+^, CH_2_O^+^	SiH_4_, NO, CH_2_O
44	CO_2_^+^, SiNH_2_^+^, SiHMe^+^	CO_2_, H_3_SiNH_2_, SiH_3_Me
45	SiNH_3_^+^	H_3_SiNH_2_
146	HNSi_2_Me_5_	(Si(CH_3_)_3_)_2_NH

a The assignment of ions is in accordance with previous studies.^4,32^

**Figure 3:**
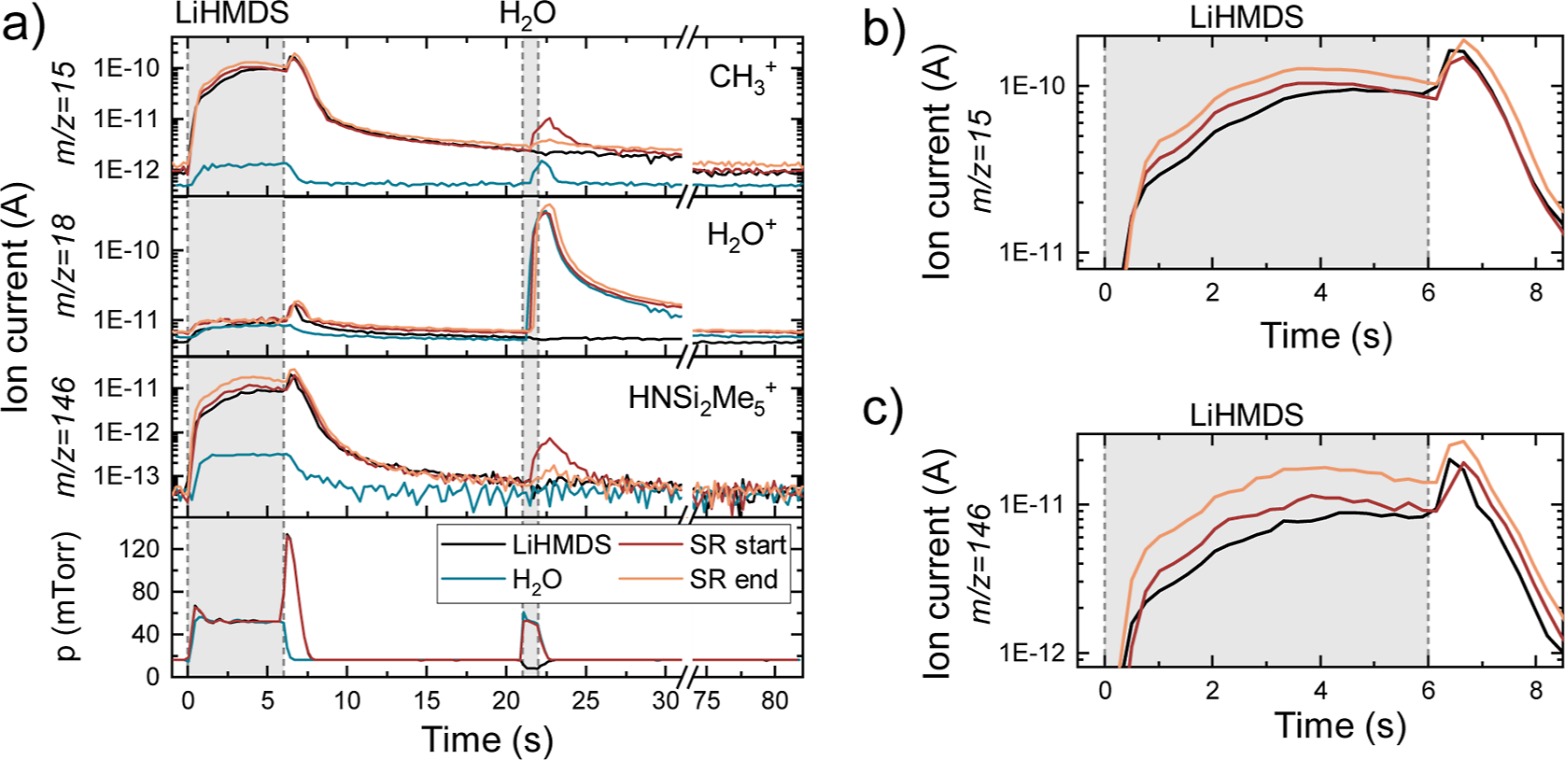
(a) Time-resolved QMS data of selected *m*/*z* values during the LiHMDS + H_2_O process. The standard ALD recipe (SR) at the start and end of the deposition, corresponding to the slow and fast growth regimes (earlier defined in Figure 1) respectively, are compared to the recipe with only H_2_O and the recipe with only LiHMDS. Zooms of the HMDS-related signals of *m*/*z* = 15 amu (b) and 146 amu (c) during the LiHMDS dose step.

Figure 3a shows the time-resolved QMS data for the LiHMDS + H_2_O process. Measurements of the SR were taken during the first and last 10 cycles of a total of 100 ALD cycles, which correspond to the slow and fast growth regimes, respectively. During the precursor dose step an increase in the signal of the fragments attributed to the HMDS ligand (*m*/*z* = 15 and 146 amu, Figure 3b,c) can be observed for the initial cycles of the SR, relative to the LiHMDS-only recipe. Since for the LiHMDS-only recipe all surfaces are expected to be saturated with the precursor, the increase in HMDS signal for the SR can be attributed to chemisorption of LiHMDS on surface OH-groups. LiHMDS is a strong non-nucleophilic base, and this drives the chemisorption by proton exchange, resulting in the formation of HMDS, in agreement with Werbrouck et al.^32^
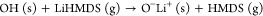
1

The formed HMDS is said to also chemisorb, resulting in Si incorporation.^31,32^ However, HMDS is less reactive toward OH-groups than LiHMDS due to differences in electronegativities^31^ and its strong preference for isolated OH-groups.^33^ Furthermore, the films grown with H_2_O as coreactant are Si-free. Therefore, we conclude that Si incorporation does not occur due to chemisorption of HMDS during the precursor dose step. The continuously supplied LiHMDS presumably dominates the reactions with the OH-groups, while HMDS is pumped away. During the fast growth regime, a stronger QMS signal for the HMDS fragments is observed than in the low growth regime (Figure 3b,c), indicating an increase in chemisorption. This is attributed to chemisorption reactions involving bulk OH-groups, as opposed to reactions with only surface OH-groups during initial cycles.

Signals of *m*/*z* = 15 and 146 amu are also detected during the H_2_O dose step at the start of the SR, indicating that HMDS is removed from the surface. This suggests that, in addition to chemisorption, physisorption also occurs during the precursor dose step, as was proposed by Werbrouck et al.^32^ They name it self-saturating dipole-driven physisorption, facilitated by the intrinsic dipole moment in the Li–N bond of LiHMDS molecule and the O–Li^+^ surface groups formed after the chemisorption of LiHMDS.
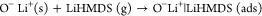
2

The physisorbed LiHMDS molecules screen the surface dipoles, such that this physisorption process is self-saturated. Steric hindrance potentially impedes chemisorption of HMDS on unreacted OH-surface sites. This mechanism of sequential chemisorption and physisorption of LiHMDS is found to be the same for all processes presented in this work.

The observed removal of HMDS ligands by reactions with H_2_O is in line with the absence of Si in the grown films. It is proposed that H_2_O acts as a weak acid, leading to a proton exchange reaction with the physisorbed LiHMDS and resulting again in an OH-terminated surface.
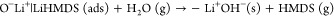
3

The HMDS signal during the H_2_O dose step shows a decrease at later cycles of the SR, compared to the initial cycles. The reduced physisorption of LiHMDS in the fast growth regime suggests a preference for (bulk) chemisorption over physisorption.

It is expected that the bulk OH-groups are regenerated during the H_2_O dose step. For example, H_2_O can react with bulk Li_2_O to form LiOH according to the following reaction
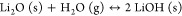
4

**Figure 4:**
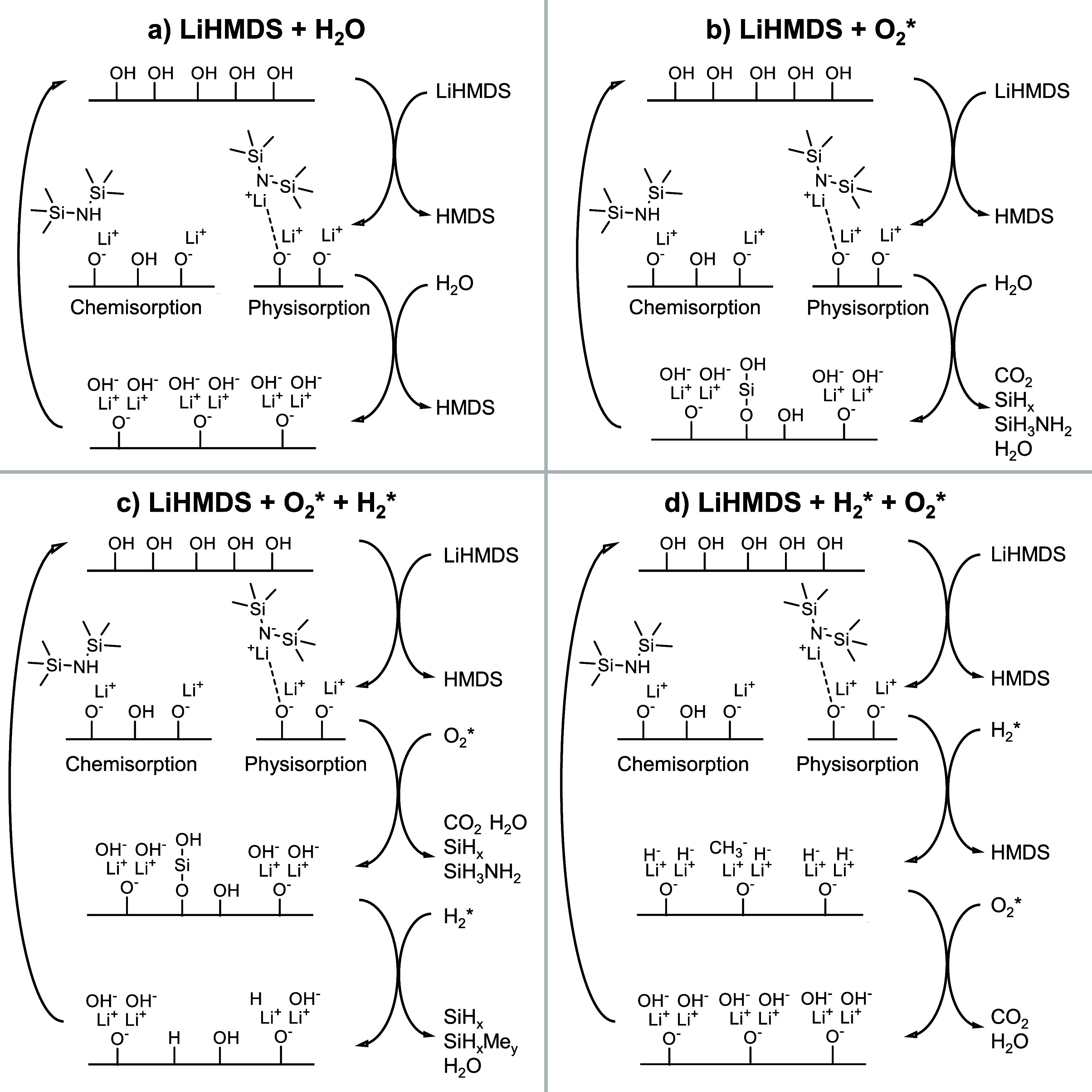
Schematics of the reaction mechanisms occurring during the (a) LiHMDS + O_2_*, (b) LiHMDS + H_2_O, (c) LiHMDS + O_2_* + H_2_*, and (d) LiHMDS + H_2_* + O_2_* processes.

Additionally, LiOH is hygroscopic and can absorb H_2_O molecules. It is expected that bulk chemisorption reactions can convert (bulk) LiOH back to Li_2_O. The overall proposed reaction mechanism for the LiHMDS + H_2_O process is schematically shown in Figure 4a.

**Figure 5:**
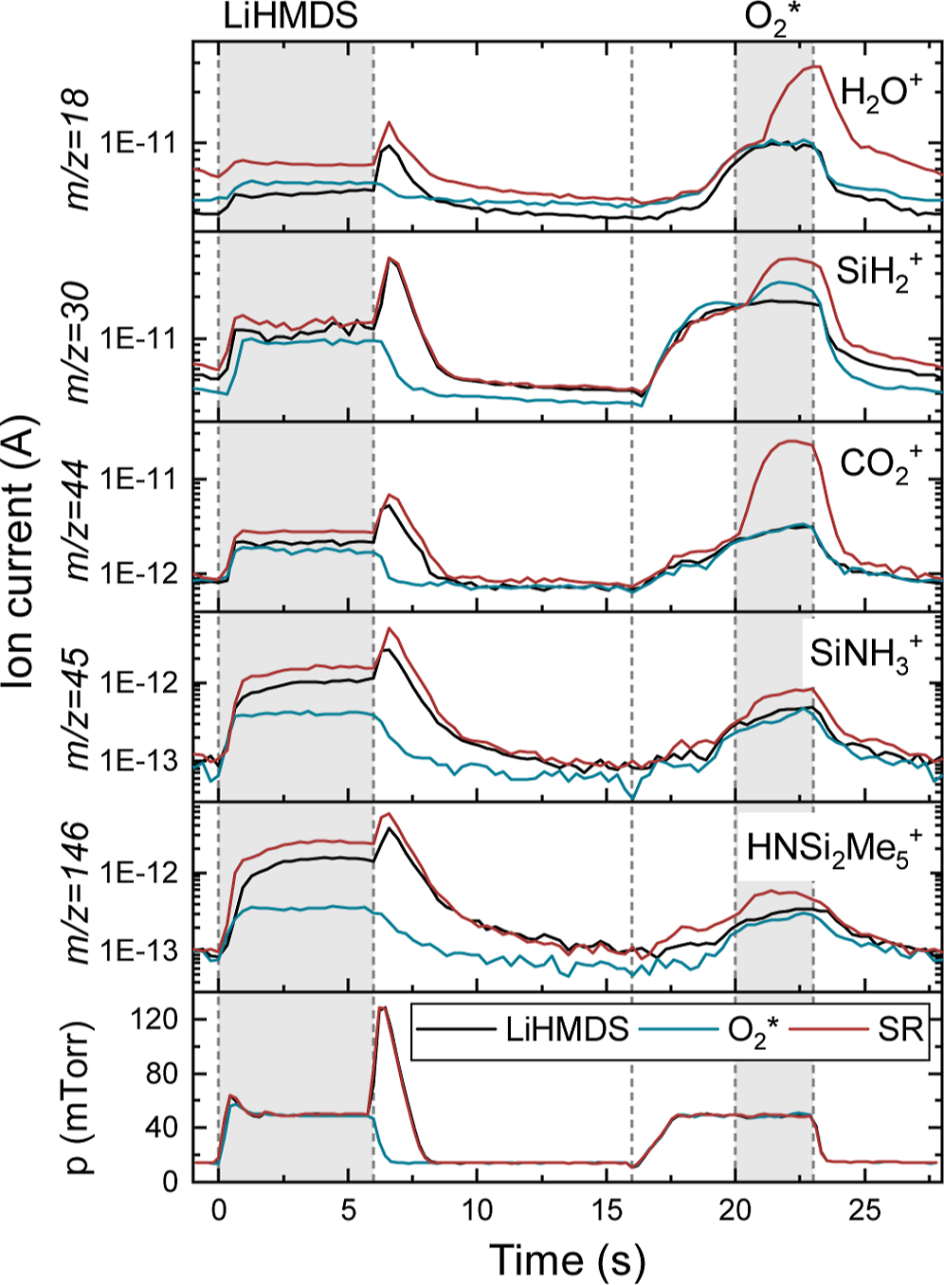
Time-resolved QMS data of selected *m*/*z* values during the LiHMDS + O_2_* process. The standard ALD recipe (SR, red) is compared to the recipe with only O_2_* (blue) and the recipe with LiHMDS and O_2_ gas (black).

The time-resolved QMS data for the LiHMDS + O_2_* process are shown in Figure 5. During the precursor dosing in the LiHMDS + O_2_* process the previously discussed chemisorption and physisorption of LiHMDS take place. Similar to the LiHMDS + H_2_O process, the chemisorption of LiHMDS is observed from the increase in HMDS signal (*m*/*z* = 146 amu) for the SR, compared to the LiHMDS-only recipe. Physisorption is observed from the ligand combustion during the O_2_* step. In addition to the expected combustion products, such as H_2_O and CO_2_ (*m*/*z* = 18 and 44 amu), Si-containing fragments are also observed, i.e., *m*/*z* = 30, 45, and 146 amu, which are attributed to SiH_2_^+^, SiNH_3_^+^, and HNSi_2_Me_5_^+^, respectively (see Table 2). Since the plasma process results in Si incorporation and the thermal process does not, Si incorporation must occur during the O_2_* step. Figure 4b sketches the proposed reaction mechanism. It is plausible that the reaction products formed in the O_2_* step are not immediately pumped away and can be redeposited on the surface of the growing film. Knoops et al. showed that the residence time of the species in the plasma, defined as the time molecules reside in the ALD reactor before being pumped away, has a strong influence on the incorporation of redeposited species in the grown film.^42^ Redeposition was shown to occur for residence times as low as 0.4 s. Using the method of Knoops et al. the residence time during the 3 s O_2_* step is calculated to be 1.1 s, which is long enough to facilitate the redeposition of Si containing fragments on the surface. It is expected that the residence time affects the Si content in the grown film. Because the residence time strongly depends on the geometry of the ALD reactor, this could be a reason for the large differences between reported Si contents for the LiHMDS + O_2_* process.^32,34^

### Si Abstraction by Means of an H_2_ Plasma

Since the thermal process exhibits non-ALD-like growth, the plasma process would be preferred for implementation in supercycle processes to fabricate ternary materials. However, this application requires a strategy to lower the Si content in the films grown with O_2_* as coreactant. With the knowledge that the O_2_* step is responsible for the Si incorporation, we hypothesized that the addition of a H_2_* step after the O_2_* step can etch the incorporated Si by producing volatile species such as SiMe_*x*_H_4–*x*_ molecules.^43^ Whereas the LiHMDS + O_2_* process shows controlled and linear growth, the addition of a 2 s H_2_* after the O_2_* to the ALD cycle (referred to as the LiHMDS + O_2_* + H_2_* process) resulted in the same type of fast growth that was observed for the LiHMDS + H_2_O process (Figure 1). Furthermore, the resulting film was capped with Al_2_O_3_ because of its reactivity toward air (Table S1). Both XPS surface measurements (Table 1) and depth profile (Figure S7) show that the LiHMDS + O_2_* + H_2_* process resulted in a Si-free film with a Li_2_O composition. This is further supported by the refractive index of 1.62 determined by *in situ* SE (Table S1), which is close to the refractive index of Li_2_O (1.64). Li_2_O readily reacts with H_2_O and CO_2_ to form Li_2_CO_3_, thus explaining the observed air-sensitivity of these films.

Because the LiHMDS + H_2_O process showed similar growth behavior, and Si-free, air-sensitive films, we conclude that the LiHMDS + H_2_O process also leads to Li_2_O films, with Al diffusion suggesting that the films have a porous structure. Contrastingly, the LiHMDS + O_2_* + H_2_* process results in denser films, as Al_2_O_3_ is only found on the film surface, instead of throughout the bulk. It is commonly observed that plasma ALD films have higher mass density than thermal ALD films due to the ion flux reaching the film surface in the plasma step(s).^44^

Additionally, the effect of changing the order of the two plasma steps has been investigated. The LiHMDS + H_2_* + O_2_* process shows the same growth behavior (Figure 1), refractive index (Table S1), and surface and bulk composition (Table 1 and Figure S8) as observed for the LiHMDS + O_2_* + H_2_* process.

**Figure 6:**
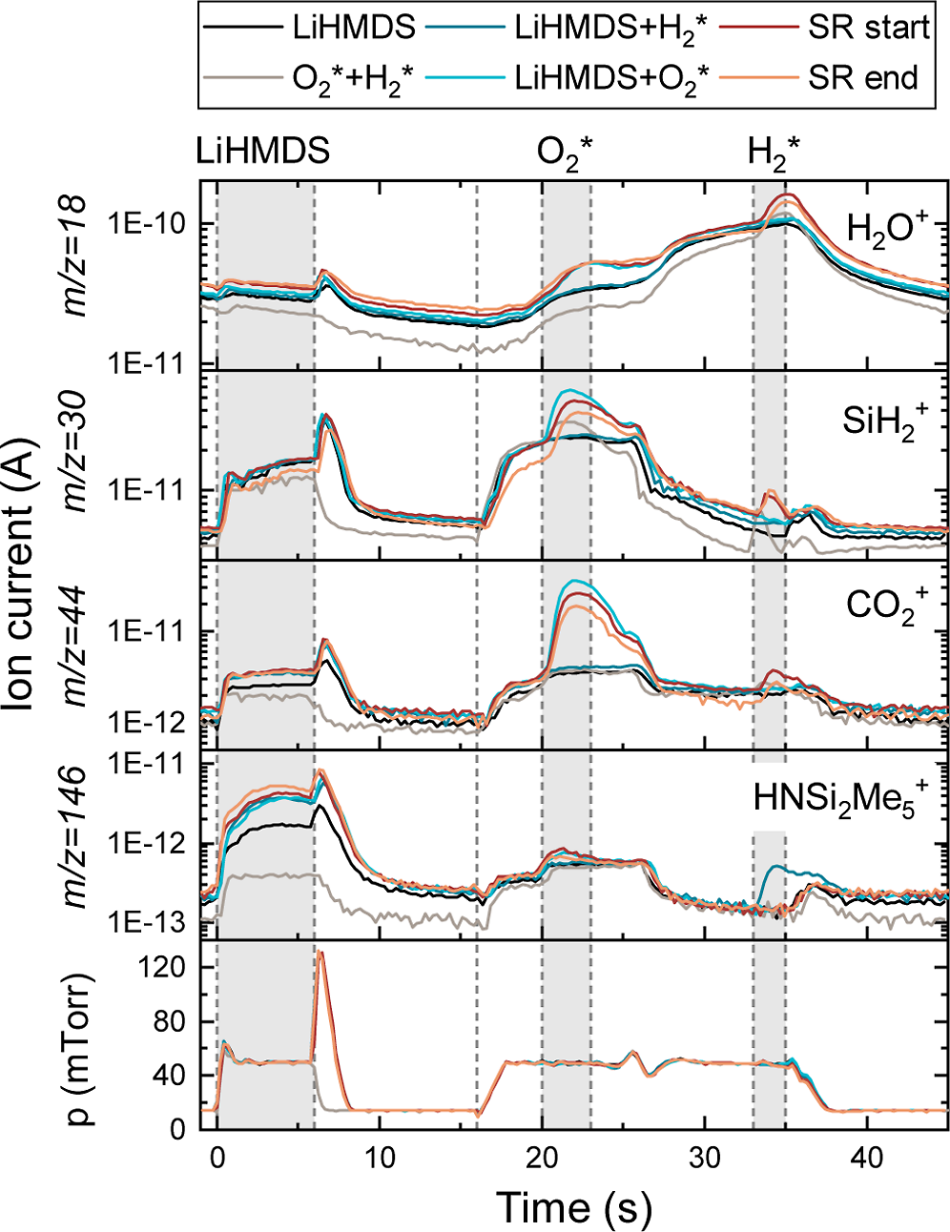
Time-resolved QMS data of selected *m*/*z* values during the LiHMDS + O_2_* + H_2_* process. The standard ALD recipe (SR) at the start and end of the deposition, corresponding to the slow and fast growth regimes respectively, are compared to the recipes with only LiHMDS, only O_2_* + H_2_*, only LiHMDS + H_2_* and only LiHMDS + O_2_*.

Time-resolved QMS measurements (see Figure 6) were performed for the LiHMDS + O_2_* + H_2_* process to test the Si-etching hypothesis. Measurements were taken during the slow and fast growth regimes, but the differences between the two regimes are less pronounced than for the thermal process. Chemisorption and physisorption during precursor dosing, as well as the ligand combustion and Si redeposition during the O_2_* step, have been described in the previous section. During the H_2_* step, a signal at *m*/*z* = 30 amu (SiH_2_^+^) is observed in the SR, confirming the hypothesis that Si is etched. The proposed reactions during this process are summarized in Figure 4c. Furthermore, H_2_O is formed in the H_2_* step, possibly due to the removal of OH-surface groups. We argue that H_2_* species and/or H_2_O formed by the plasma are able to remove the entire HMDS ligands of physisorbed LiHMDS from the surface, which can explain the observed signal at *m*/*z* = 146 amu during the H_2_* step of the LiHMDS + H_2_* recipe.

**Figure 7:**
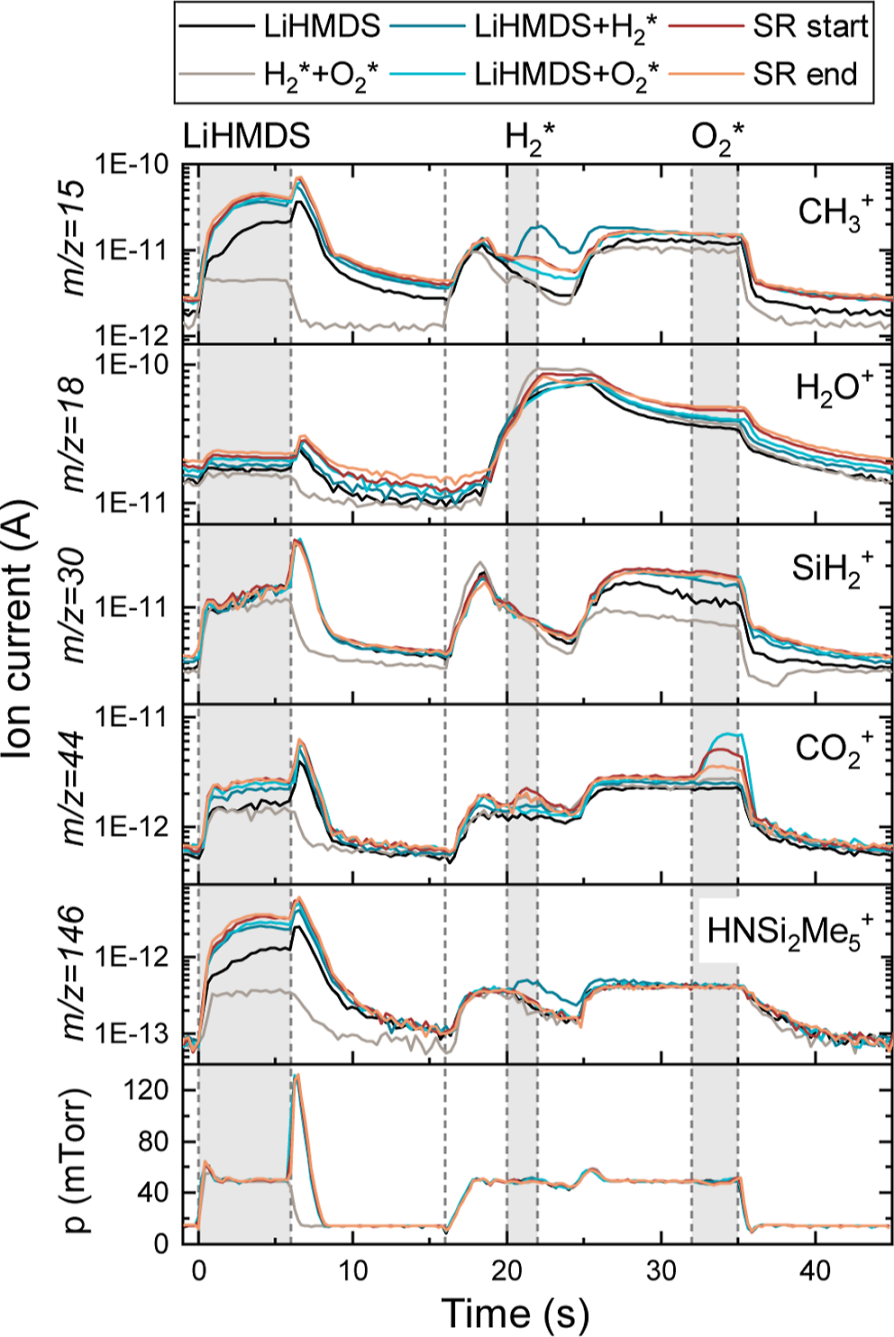
Time-resolved QMS data of selected *m*/*z* values during the LiHMDS + H_2_* + O_2_* process. The standard ALD recipe (SR) at the start and end of the deposition, corresponding to the slow and fast growth regimes respectively, are compared to the recipes with only LiHMDS, only O_2_* + H_2_*, only LiHMDS + H_2_* and only LiHMDS + O_2_*.

Also the LiHMDS + H_2_* + O_2_* process was investigated using time-resolved QMS, as shown in Figure 7. This process has shown poor reproducibility, in particular regarding species detected during the H_2_* step. Similar to the other processes, during the LiHMDS dose chemisorption and physisorption occur. Other measurements of the same process presented in Figure S9 show clear signals at *m*/*z* = 15 and 146 amu during the H_2_* step, which are not visible in Figure 7. As proposed above, the H_2_* appears to remove the HMDS ligands and thereby prevent the incorporation of Si in the subsequent O_2_* step. Additionally, combustion products, CO_2_ in particular (*m*/*z* = 44 amu), are observed during the O_2_* step. This suggests the presence of carbon-containing species on the surface after the H_2_* step. These species are likely fragments of the physisorbed LiHMDS molecules, but their type has not been determined. The decrease in the *m*/*z* = 44 amu signal from the start to the end of the SR is in line with the proposed preference of (bulk) chemisorption over physisorption, resulting in less physisorbed LiHMDS during later ALD cycles. Furthermore, it was observed that the LiHMDS + H_2_*+O_2_ (gas) process still results in high growth rates, suggesting that H_2_O formed during the H_2_* steps plays a crucial role in the film growth by forming bulk OH-groups, similar to the LiHMDS + H_2_O process. A schematic of the proposed surface reactions is shown in Figure 4d.

## Conclusion

LiHMDS was combined with O_2_* and H_2_O as coreactants to investigate the origin of its dual-source behavior. Linear growth was observed for the LiHMDS + O_2_* process, which results in a film that contains 16.2 at. % Si. The LiHMDS + H_2_O process on the other hand showed fast, non-surface-reaction-limited growth and yielded Si-free, but air-sensitive films. Time-resolved QMS measurements were performed to gain insight on the effect of the coreactant on the reaction mechanisms of LiHMDS. The increased HMDS signal during the LiHMDS dose step in the SR compared to the coreactant-only processes, is attributed to a ligand exchange reaction between surface OH-groups and LiHMDS molecules. The fast growth observed for the LiHMDS + H_2_O process is attributed to LiHMDS chemisorption involving bulk OH-groups. The observation of HMDS molecules and HMDS combustion products during the H_2_O and O_2_* steps, respectively, indicates that LiHMDS also physisorbs on the surface of the film. Redeposition of Si-containing combustion products during the O_2_* step is determined to be the origin of Si incorporation into the grown film.

The effect of adding an H_2_* step to the O_2_*-based process was investigated as a strategy to obtain Si-free films. The growth behavior and film composition were similar to the LiHMDS + H_2_O process, independent of the order of the H_2_* and O_2_* steps. QMS measurements showed that the H_2_* step in the LiHMDS + O_2_* + H_2_* process etches Si, that was incorporated in the film in the previous O_2_* step. When applied directly after the LiHMDS dose, the H_2_* presumably removes the ligands of physisorbed LiHMDS, which prevents Si incorporation in the film during the O_2_* step.

The QMS study presented in this work provides evidence for the sequential chemisorption and physisorption reaction mechanism of LiHMDS, which was previously proposed in literature. However, reactions between HMDS and surface OH-groups were found to not play a significant role in the Si incorporation in the film. Instead, it is shown that the choice of coreactant determines whether LiHMDS acts as a dual- or single-source. These insights on the reaction mechanism of LiHMDS and the role of the coreactant on its dual-source behavior may help the development of LiHMDS-based ALD processes for Li-ion battery applications.

## Data Availability

The data underlying this study are openly available in the 4TU.ResearchData repository at 10.4121/06bf96d9-18a7-4738-a1d8-efa8abbc7154.

## Supporting Information Available

Additional information on the origin of F surface contamination, saturation curves of the LiHMDS + O_2_* process at 200 °C, XPS Si 2p surface scan, XPS depth profiles, *in situ* and *ex situ* SE results, photos of air-sensitive films, a zoom of the LiHMDS mass spectrum showing a peak at *m*/*z* = 174 amu, and repeated time-resolved QMS measurements of *m*/*z* = 146 amu in the LiHMDS + H_2_* + O_2_* process (PDF)
