# Supplementary material for: Role of the Coreactant on the Dual-Source Behavior of Lithium Hexamethyldisilazide for ALD Li-Containing Films
Source: J Phys Chem C Nanomater Interfaces. 2024 Nov 7;128(46):19638–47. doi: 10.1021/acs.jpcc.4c05987 (PMC11587086; doi:10.1021/acs.jpcc.4c05987)
Supplement: Supplementary file 1 — jp4c05987_si_001.pdf [file jp4c05987_si_001.pdf]

Supporting information for

Role of the Coreactant on the Dual-Source Behavior of Lithium Hexamethyldisilazide for ALD Li-Containing Films

M.J. Pieters[1,*], L. Bartel[1], C. van Helvoirt[1], M. Creatore[1,2]

[1] Department of Applied Physics and Science Education, Eindhoven University of Technology, 5600 MB, Eindhoven, The Netherlands

[2] Eindhoven Institute of Renewable Energy Systems (EIRES), PO Box 513, 5600 MB Eindhoven, The Netherlands

* Email: m.j.pieters@tue.nl

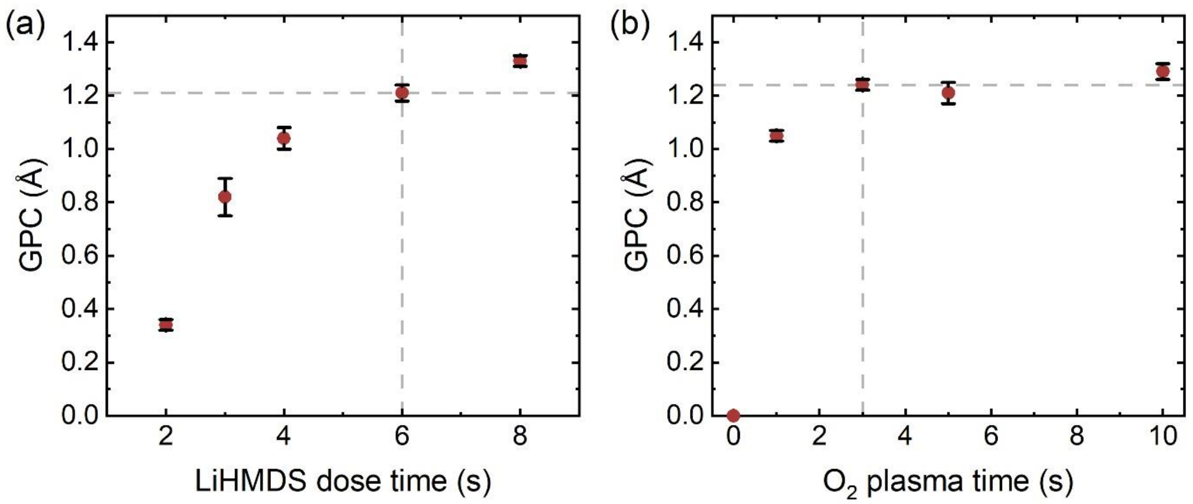

Figure S1 – Saturation curves of the LiHMDS+O_2 ALD process at 200°C. During the variation of the LiHMDS dose time, the O_2* time was kept at 5 s. During the variation of the O_2* time, the LiHMDS dose time was kept at 6 s.*

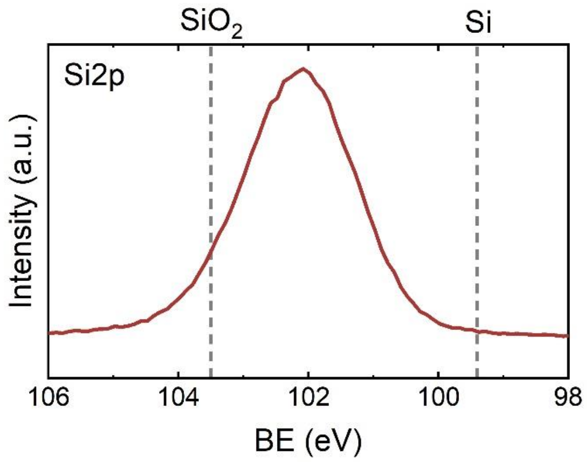

Figure S2 – XPS Si2p spectrum of the film grown using the LiHMDS+O_2 process.*

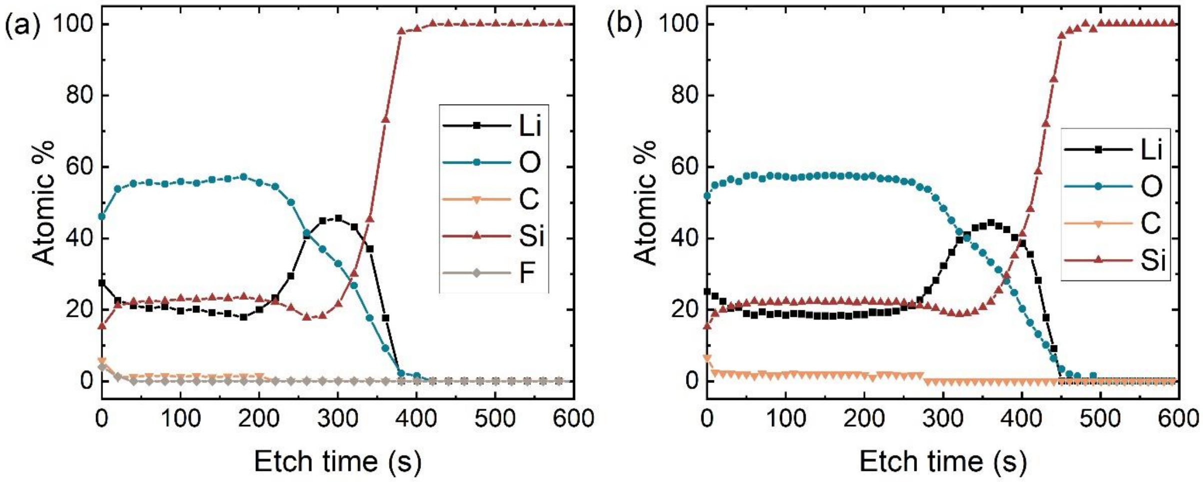

Figure S3 - XPS depth profile of films grown using the LiHMDS+O_2 process at deposition temperatures of 200°C (a) and 120°C (b). The film thicknesses are 30 and 23 nm respectively. No F was detected on the surface of the film deposited at 120°C.*

Origin of F surface contamination

The F surface contamination observed by XPS on most of the Li-containing films in this work originates from the scroll pump that is connected to the loadlock of the ALD reactor. Therefore, the scroll pump was replaced by a multi-stage roots pump, but only after most of the deposition that are part of this work were performed. The deposition of the LiHMDS+O_2* at 120°C (see Figure S3b) was done after the pump replacement and no F was detected on the film surface. Because the F surface contamination is linked to the loadlock and not the ALD deposition chamber, it is independent from the ALD growth mechanisms that are investigated in this work.

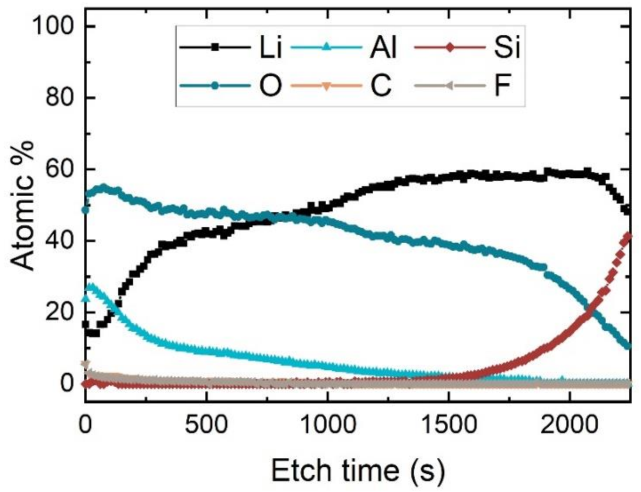

Figure S4 – XPS depth profile of a 117 nm film grown using the LiHMDS+H_2O process. The etch time was not long enough to fully reach the Si substrate.

Table S1 – Comparison of thicknesses of uncapped films determined from in situ and ex situ SE measurements. The mean square error (MSE) of the fits are shown as a measure of the quality of the fits, which provided the film thickness and refractive index.

	***In situ* SE**			***Ex situ* SE**		
	Thickness (nm)	n@1.96 eV	MSE (a.u.)	Thickness (nm)	n@1.96 eV	MSE (a.u.)
LiHMDS + H_2O	56.3	1.51	11	126	1.36	45
LiHMDS + O_2*	24.6	1.44	5	29.4	1.52	5
LiHMDS + O_2* + H_2*	49.2	1.62	10	128	1.43	30
LiHMDS + H_2* + O_2*	48.3	1.64	12	171	1.41	73

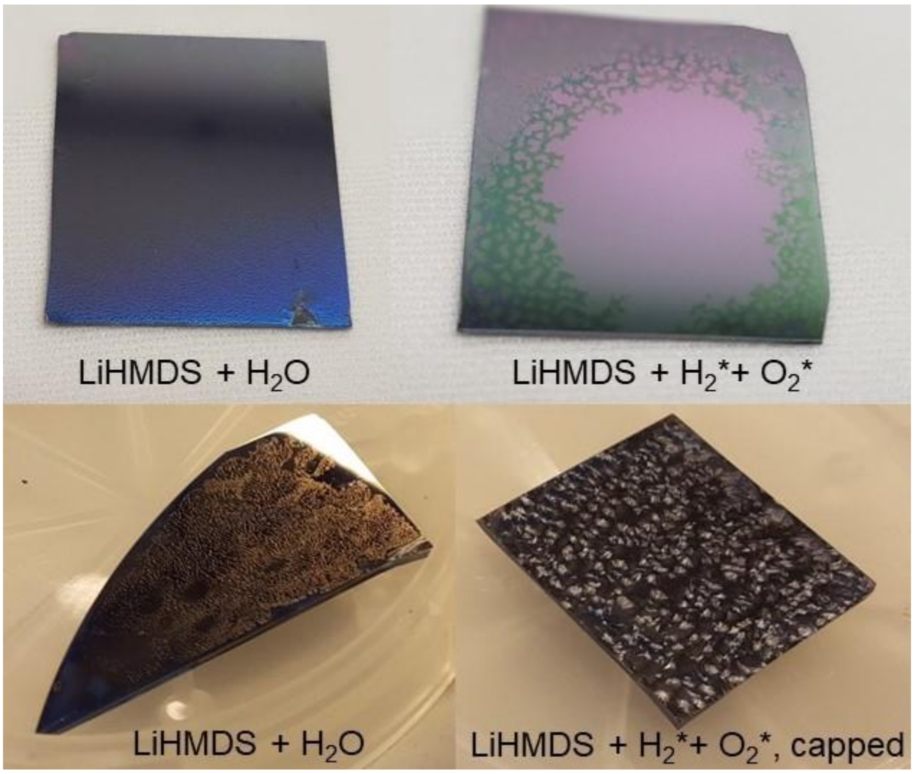

Figure S5 – Photos of several air sensitive films that have been stored in air. The effect of air exposure on the appearance of the film differs from sample to sample. The ~5 nm Al_2O_3 capping layer is not effective in protecting the underlying film against prolonged air exposure.

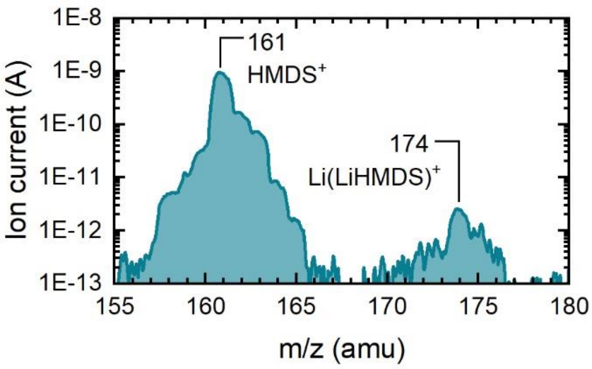

Figure S6 – Zoom of the LiHMDS mass spectrum on a logarithmic scale.

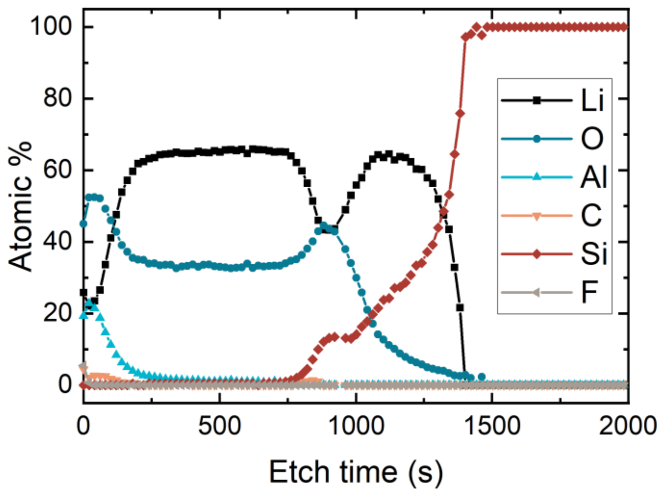

Figure S7 – XPS depth profile of a 70 nm film grown using the LiHMDS+O_2+H_2* process.*

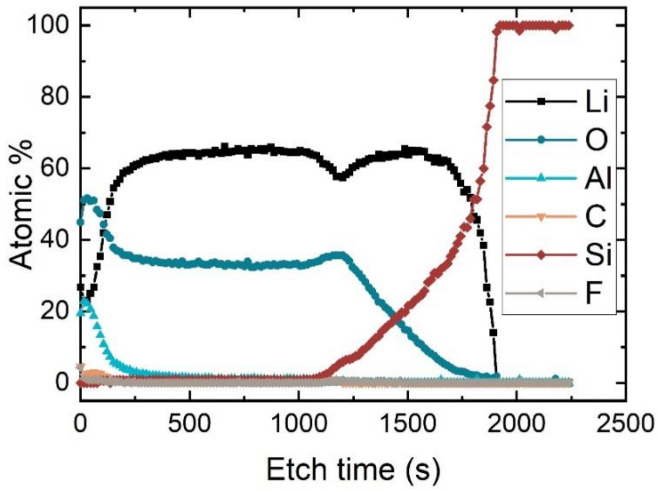

Figure S8 – XPS depth profile of a 90 nm film grown using the LiHMDS+H_2+O_2* process.*

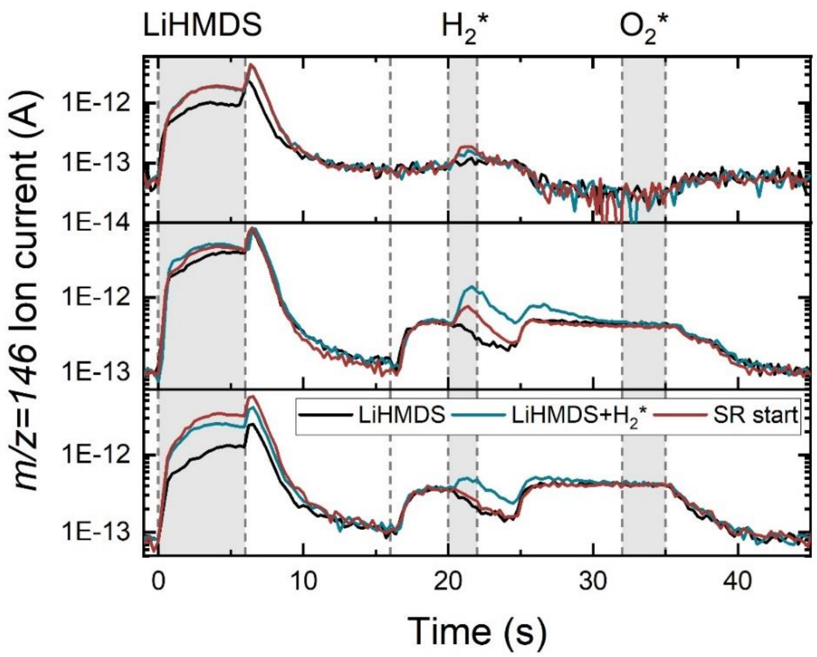

Figure S9 – Comparison of m/z=146 amu QMS signal during three separate measurements of the LiHMDS+H_2+O_2* process. Differences between the signals during the H_2* step could be ascribed to differences in the reactor wall conditions.*
